# Nanocrystal Compressive Residual Stresses: A Strategy to Strengthen the Bony Spines of Osteocytic and Anosteocytic Fish

**DOI:** 10.1002/advs.202410617

**Published:** 2025-04-11

**Authors:** Andreia Silveira, Anton Davydok, Christina Krywka, Mario Scheel, Timm Weitkamp, Claudia Fleck, Ron Shahar, Paul Zaslansky

**Affiliations:** ^1^ Department for Restorative Preventive and Pediatric Dentistry Charité‐Universitaetsmedizin 14197 Berlin Germany; ^2^ Institute of Materials Physics Helmholtz‐Zentrum Hereon 21502 Geesthacht Germany; ^3^ Synchrotron SOLEIL Saint‐Aubin 91190 France; ^4^ Fachgebiet Werkstofftechnik/Chair of Materials Science & Engineering Institute of Materials Science and Technology Faculty III ‐ Process Sciences Technische Universität Berlin 10623 Berlin Germany; ^5^ Koret School of Veterinary Medicine The Robert H. Smith Faculty of Agriculture Food and Environmental Sciences Hebrew University of Jerusalem Rehovot 76100 Israel

**Keywords:** anosteocytic and osteocytic bone material, nanocomposite compressive residual stresses, nanobeam diffraction/fluorescence imaging

## Abstract

Bone is a living tissue in which communicating cells, osteocytes, are assumed to be vital for tissue turnover and adaptation. Interestingly however, most advanced teleost fish do not possess osteocyte‐mediated porosity, prompting intriguing questions about alternative material‐strategies for these bones to cope with damage. Using advanced imaging techniques, including phase‐contrast enhanced (PCE) microtomography (µCT) and nanotomography (nanoCT), X‐ray fluorescence (XRF), and diffraction (XRD) tomography, the micro‐ and nano‐architectures of osteocytic zebrafish are compared with anosteocytic medaka fishbone. PCE µCT and Zernike phase‐contrast nanoCT showed a lack of porosity in medaka bone and 0.75 – 2.26% osteocytic porosity in zebrafish. Both fish species have similar mineralized collagen fibril arrangements containing calcium (Ca) and traces of strontium (Sr) with increased zinc (Zn) localized on the outer bone regions. Medaka bones exhibit reduced apatite nanocrystal lattice spacings on the outer surfaces. Indeed we find higher compressive residual strains (‐0.100 ± 0.02) compared to zebrafish (‐0.071 ± 0.03). We propose that medaka bone evolved to replace the mechanosensitive osteocytic network by entrapping protective residual strains between collagen nanofibers and mineral crystals. These strains may enhance fracture toughness while making this nanocomposite well‐suited for sustaining repeated loading cycles, thus reducing the metabolic costs associated with housing a large network of cells.

## Graphical abstract

Advanced neoteleost fishbones, such as medaka, challenge bone adaptation strategies. While zebrafish bones contain osteocyte‐mediated porosity, medaka bones lack it, raising questions about alternative reinforcement mechanisms. Using advanced imaging, this study reveals higher residual compressive strains in medaka bone, suggesting an adaptation that enhances fracture toughness. These insights offer a new perspective on how bone material compensates for osteocytes absence.

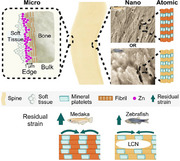

## 1 Introduction

Bones are “smart” biogenic structures mainly made of mineralized collagen fibrils and they are able to adapt and meet the demands of locomotion and mechanical load‐bearing.^[^ ^1^, ^2^, ^3^ ^]^ However, it is not fully understood how the properties of the bone nanocomposite complement biological processes that change the structure to meet environmental stresses.^[^ ^4^ ^]^ Recent studies have highlighted very different strategies of structural adaptation within the skeletal tissues of anosteocytic medaka (*Oryzias latipes*) and osteocytic zebrafish (*Danio rerio*) bones.^[^ ^5^, ^6^, ^7^ ^]^ Zebrafish bones are characterized by an osteocytic lacuno‐canalicular network (LCN) similar to the one found in mammalian bones. This network facilitates mechanosensation and the required coordinated responses to mechanical stimuli that regulate bone remodeling.^[^ ^8^ ^]^ On the other hand, anosteocytic bone, which is found in most neoteleost fish, including medaka, challenges traditional paradigms of bone physiology and mechanotransduction, as it is completely devoid of osteocytes and their associated LCN.^[^ ^9^, ^10^, ^11^ ^]^ In the absence of such cells, anosteocytic bones rely on alternative mechanisms to perceive and respond to changes in loading conditions.^[^ ^5^, ^6^ ^]^ While the exact mechanisms remain elusive, Ofer et al. hypothesized that the SOST gene is expressed by a variety of nonosteocytic cells that mediate bone remodeling.^[^ ^5^, ^6^ ^]^ The lack of osteocytes within these bones raises questions regarding the importance of bone mechanosensation as a means to prevent or recover damage from overload.

For osteocytic bones to communicate external loads across the LCN, the signal must flow effectively. A widely accepted theory, known as the bone poroelasticity model, suggests that deformation information is delivered through fluid pressure gradients inside the LCN.^[^ ^4^, ^12^, ^13^ ^]^ This model implies that fluid must be kept within the network. Recent work has shown that proteoglycans, known to retain water, are far less abundant in the bone matrix of medaka as compared to zebrafish.^[^ ^14^ ^]^ Water flow in zebrafish bone appears to be confined to the LCN network, suggesting that osteocytic bone may have a mechanism regulated by the proteoglycans that modulates the permeability of the bone matrix.^[^ ^14^ ^]^ On the other hand, in anosteocytic medaka bone, most of the water is free to flow according to pressure gradients.^[^ ^14^ ^]^ This raises the possibility that the bone matrices of the two species are fundamentally different, not just because of the presence or absence of osteocytes. Such contrasts highlight the remarkable diversity of skeletal adaptations across vertebrate species. Understanding the functional implications of these differences may provide valuable insights into the fundamental principles governing bone biology and adaptation to diverse environmental conditions.

Over the past decades, modern X‐ray imaging methods have become indispensable in bone research, offering insight into the morphological intricacies of skeletal tissues. In particular, µCT and nanoCT have been used to elucidate the spatial relationships between the cells and mineralized matrix within bone.^[^ ^15^, ^16^, ^17^ ^]^ To truly grasp the subtle nuances of bone tissue, PCE techniques are particularly useful to reveal fine structural details. PCE exploits coherence, generating contrast from phase shifts in the X‐rays that pass through different types of tissue, or different material densities.^[^ ^18^, ^19^ ^]^ One such high‐resolution PCE method, Zernike phase contrast imaging, has not often been successfully used for bone research (due to the formation of artifacts, such as halo‐ and shade‐off distortions now resolvable, e.g., with Deep Learning approaches^[^ ^20^ ^]^), despite having huge potential to extract important information regarding bone micro‐ and nanostructures.

When considering the atomic length‐scale, XRD tomography is a powerful tool for probing the crystalline architecture of bone. By analyzing diffraction patterns arising from the interactions between X‐rays and the carbonated apatite mineral, it is possible to obtain information about the crystal lattice spacing (d‐spacing), orientation, and size.^[^ ^21^, ^22^, ^23^ ^]^ XRD offers complementary insights into the contributions of bone mineral to mechanical performance.^[^ ^24^, ^25^, ^26^ ^]^ By examining changes in lattice spacing in response to load, relative to an unstressed state, XRD can uncover the presence of residual strains within the mineral nanocrystals, shedding light on how bone resists mechanical damage.^[^ ^27^, ^28^ ^]^ This analysis can uncover how bone structure responds to varying conditions, such as changes in temperature or applied stress.^[^ ^29^, ^30^, ^31^ ^]^ Residual strain is a concept that is often applied in engineering materials, such as those used in aerospace components, to ensure safety and reliability by enhancing fracture toughness and thereby delaying crack initiation and propagation.^[^ ^31^, ^32^ ^]^ Coupling XRD with XRF imaging yields information about elemental composition, providing invaluable information regarding the spatial distribution of key constituents such as Zn within bone tissue.^[^ ^33^, ^34^, ^35^ ^]^

In this work, we combine all of the above to unravel hidden nanocomposite differences between medaka and zebrafish bones to explore the interplay between structure, composition, and mechanical function. We used a combination of PCE µCT and nanoCT to investigate the structural organization of these bones. Mineral crystalline characteristics are analyzed through XRD imaging and tomography, which reveals lattice spacing variations from which we infer residual strain distributions within the bone matrix. XRF imaging adds detailed elemental mappings of Ca, Sr, and Zn. Together, these material characterization techniques allow us to dissect the interplay between bone structure and composition in anosteocytic and osteocytic bones that may have profound implications for understanding bone health and diseases.

## 2 Results and Discussion

### 2.1 Sub‐mm Morphological Characteristics of the Bones

**Figure 1:**
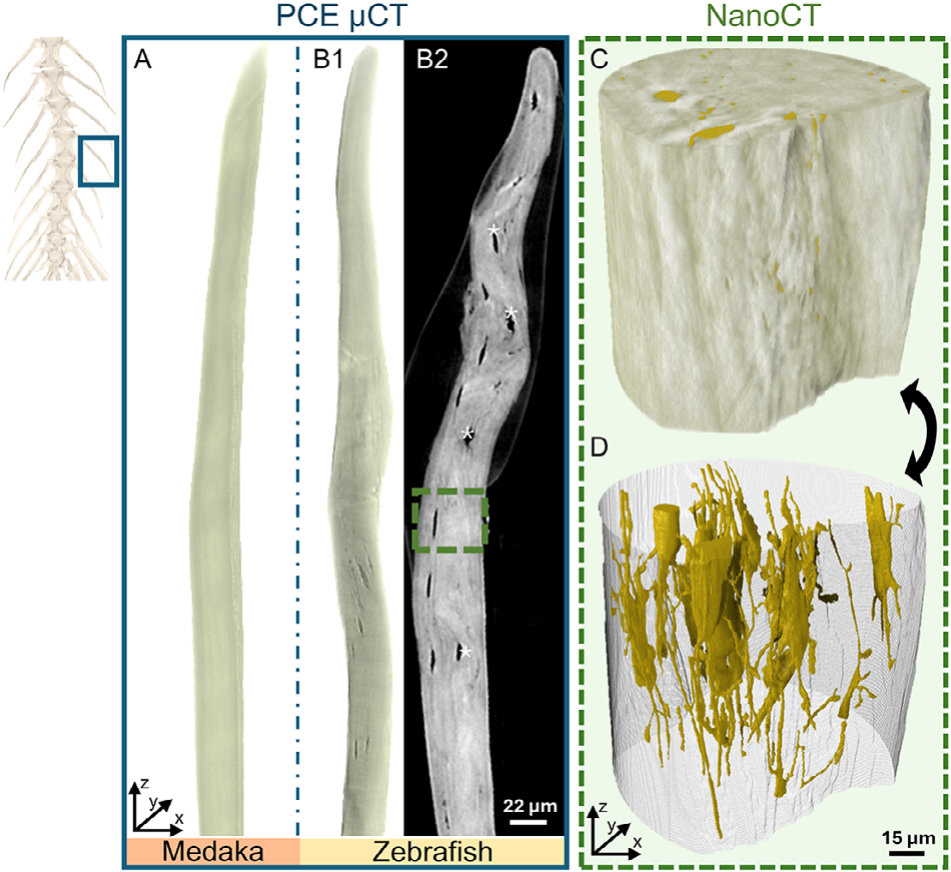
3D renderings of a spine (taken from a whole vertebral column, as indicated by upper left inset) from a PCE µCT reconstructed volume of (A) medaka and (B1) zebrafish. (B2) Example of a longitudinal slice in zebrafish bone shows voids (lacunae, marked with white asterisks). The region marked in green was imaged with Zernike‐nanoCT at higher resolution and registered for co‐alignment of the two datasets. C) a 3D rendering of the marked zebrafish bone region. D) Zernike‐nanoCT imaging reveals details of the extensive canaliculi porosity and LCN structure.

**Figure 2:**
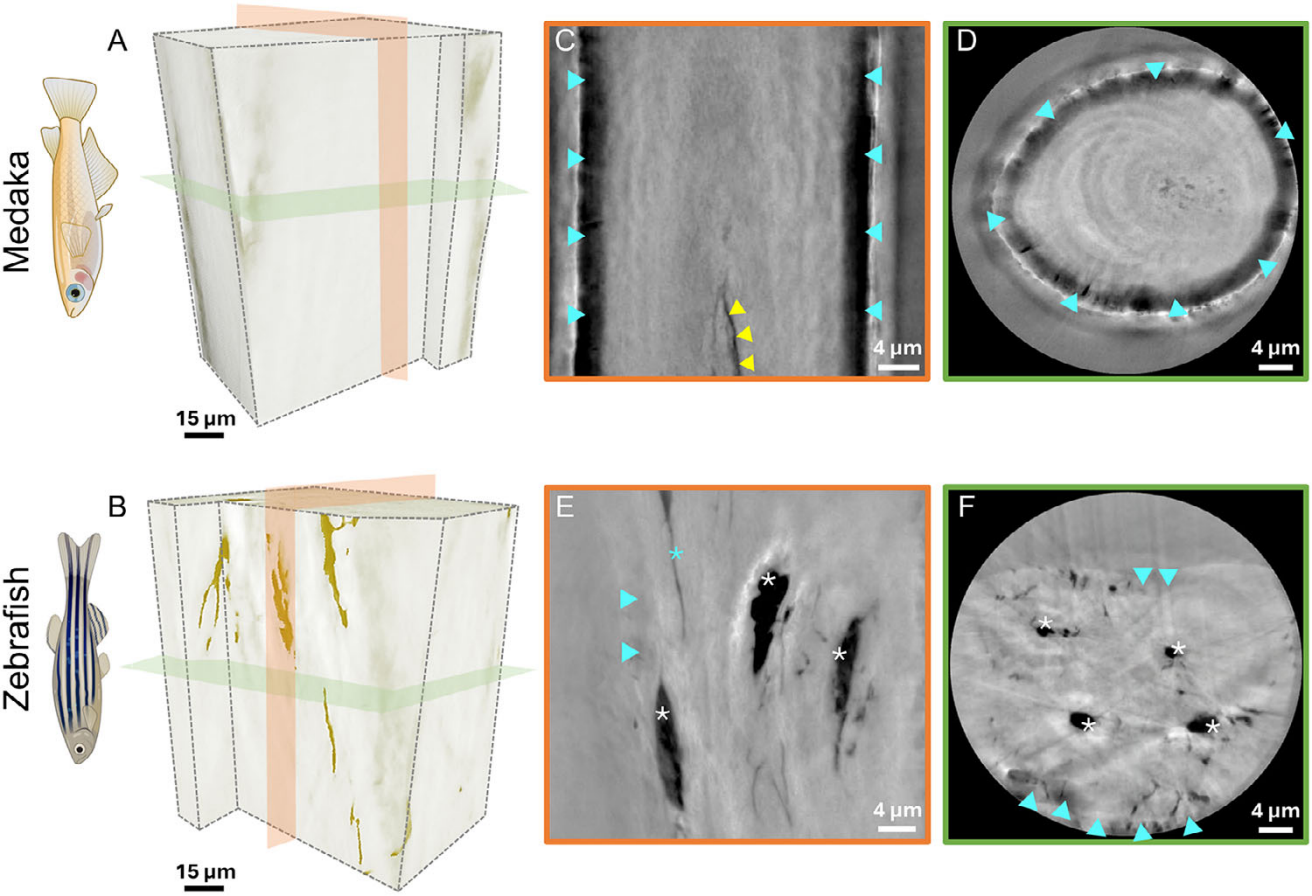
Schematic and tomographic overviews of (A) medaka and (B) zebrafish bone from Zernike‐nanoCT reconstructed data. C) Longitudinal and D) transverse sections show the compact bone layout of medaka bone hinting to the presence of aligned layers of bundles of collagen fibers with slight differences in density.^[^ ^36^ ^]^ Shade‐off and halo artifacts (marked by blue triangles) dominate the outer edges of the bone. Cracks (yellow triangles) within medaka bone are visible and are related to drying that was required for imaging. E) In the longitudinal and transverse F) sections of zebrafish bone it is easy to identify the elongated LCN voids (white asterisks), in which osteocytes reside in the living bone. Additionally, smaller voids of the canaliculi (blue asterisks) are also seen. The shade‐off artifacts (blue triangles) are less dominant in the larger diameter zebrafish bone as compared to medaka bone.

All PCE µCT scans reveal that the spines of both fish species exhibit similar geometries. **Figures** **1**A,B1 show 3D bone renderings of medaka and zebrafish spines imaged along the entire length, with data collected at an effective detector pixel size of 325 nm (Video S1, Supporting Information showcases a zebrafish spine). The main visible difference between the two fish species is the complete absence of internal lacunar voids and submicron porosity in medaka bones. The lacunae sites along the zebrafish spines are clearly visible, distributed within the bone tissue. The LCN porosity of zebrafish is shown in greater detail in a 2D slice (Figure 1B2) and can be precisely calculated from the 3D data. The LCN porosity spans 0.75 – 1.93% between different zebrafish spines. The zebrafish spine region marked by the dashed green rectangle was further scanned with Zernike‐nanoCT (Figure 1C) and the LCN structure is revealed in the 3D rendering of Figure 1D (Video S2, Supporting Information gives a 3D overview of one of the LCN renderings). To precisely locate the nano resolution scan and the LCN porosity within the PCE µCT data, 3D image correlation of the corresponding datasets was used. This helped reveal that, at different scan locations, the porosity percentage is not constant. Table S1 (Supporting Information) lists a comprehensive quantitative assessment of each lacuna and the connected canaliculi porosity revealed in 3D by Zernike‐nanoCT. The 3D bone renderings (**Figures** **2**A,B) obtained from the Zernike‐nanoCT data show that medaka bone has a compact structure while zebrafish bone contains an interconnected network of micron‐sized voids and nanochannels within the bony matrix.

### 2.2 Bone Texture and Nanocomposite Multi‐Length Scale Orientations

**Figure 3:**
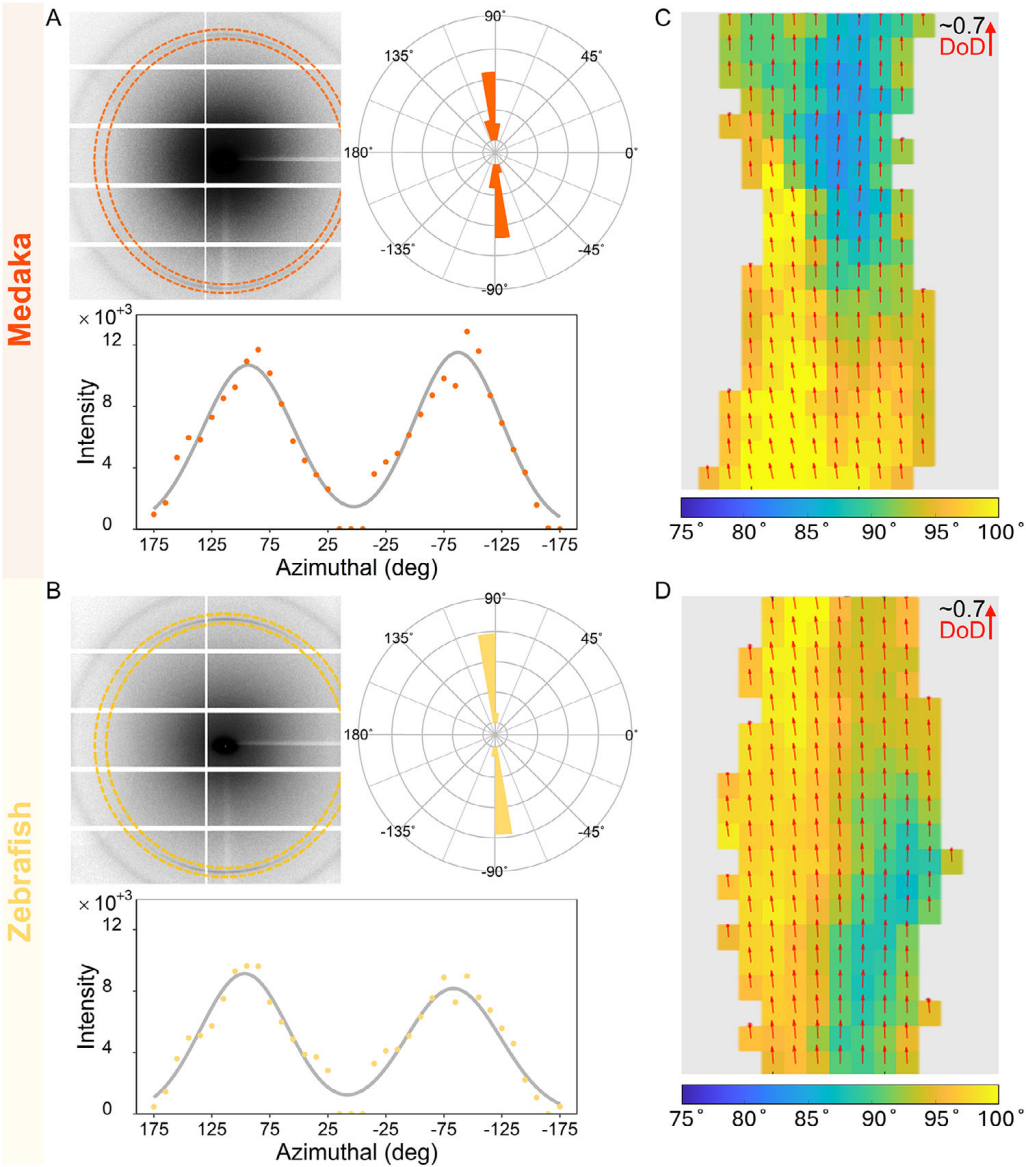
Representative XRD patterns of (A) medaka and (B) zebrafish bones. The 002 diffraction peaks were radially integrated to produce 1D azimuthal intensity profiles for each scanned point. The azimuthal intensity plots show the measured intensity (orange or yellow points) at 36 different azimuthal (χ) angles and the associated fitted line (gray color). A double‐peak Gaussian function was used to create the fitted line, which gives the peak position and provides insight into the crystal orientation. The preferred orientation for all scanned points is highlighted in a circular plot, where 95° and −85° azimuths have the highest counts for both medaka and zebrafish scans. Examples of (C) medaka and (D) zebrafish sinograms showing the mean orientation of hydroxyapatite crystals with red arrows and colorbar scale displaying the range of orientation angles for each scanned point. The lengths of the red arrows indicate the degree of preferred orientation (DoD) and has average of 0.7 score for both medaka and zebrafish.

In medaka bone material, the mineralized collagen fibrils are bundled into layers that are oriented parallel to the principal axis of the bone. Longitudinal and cross‐sectional slices in the nanoCT data (Figure 2C,D) also reveal cracks within the dry‐imaged medaka tissue that were likely caused by the high stresses induced by collagen shrinkage during dehydration. Unlike medaka, zebrafish bone (Figure 2E,F) reveals a porous network within the mineralized collagen matrix. The lacunae and canaliculi voids (marked by white asterisks) make it difficult to identify the fibril orientation although layers of varying density appear to be oriented along the principal axis of the spines. The challenge of identifying bone fiber orientation by nanoCT may also arise from the possibility of superimposing bone features onto Zernike imaging artifacts, which further complicates the analyses. Videos S3 and S4 (Supporting Information) showcase the bone ultrastructures of medaka and zebrafish. The nanoscale texture of the bones becomes easier to identify based on XRD data. Examples of 2D diffraction images reveal well‐defined 002 Debye arcs of the apatite mineral that is aligned with the collagen fibers, along the principal bone axis. The intensity of the 002 diffraction peak varies with the azimuth angle for both bone types, as demonstrated by radially integrated profiles shown in **Figure** **3**A,B. The presence of two peaks in the azimuthal intensity profiles indicates a preferred orientation of the apatite nanocrystals. If the crystals were randomly oriented, the diffracted intensity would be constant for all azimuthal angles. In both medaka and zebrafish, the highest intensity is at 95° and −85° azimuths, suggesting a preferred orientation of the c‐axis that is aligned with the longitudinal axis of the bones, matching the orientation of the collagen fibrils. By analyzing the orientation in lines obtained from different rotation angles, we obtain sinograms shown in Figure 3C,D, that reveal the preferred nanocrystal orientation for each XRD scanned angle. The orientation is indicated by colors and arrows with some points showing a deviation of up to 10° away from the principal bone axis. The degree of preferred orientation within the bone is specified by the length of the arrows (0 for no preferential orientation and 1 for high orientation) and it is consistent across most of the sinograms, with an average of ≈0.7, indicating a high degree of orientation.

The orientation ratios of the nanocrystals along the bone axis were assessed within reconstructed slices at small and large angles relative to the main axis of bone spines: 0° (parallel to the main axis), 70°/110° (<20° to main axis), and 40°/140° (<50° to main axis), see Figure S1 (Supporting Information). Examples of the reconstructed slices, including the intensities along the main axis (0°) and ratios (0°/20° and 0°/50°), are provided in Figure S2 (Supporting Information). Both medaka and zebrafish bones reveal that most nanocrystals align predominantly with the principal bone axis, as shown by the brighter regions (higher intensity) observed in the reconstructed slices of the 0° angle. It is likely that this texture makes the bones stiffer and stronger along the principal bone axis. Analyzing bone material, Currey et al. reported the orientation dependence of Young's modulus and tensile failure properties of a highly oriented mineralized tissue in the narwhal tusk.^[^ ^37^ ^]^ That study showed that mechanical properties, such as Young's modulus and strength, decreased as the angle of loading deviated from the fiber direction of bone. Seto et al. also showed that bone is much stronger and stiffer along fiber directions.^[^ ^38^ ^]^ For bone to withstand mechanical stress effectively, the alignment of fibrils orientation often matches the main loading direction. The nanocrystal c‐axis is aligned with the collagen fibril axis, and the XRD observations are consistent with the arrangements observed in the nanoCT data. The absence or presence of LCN porosity does not seem to affect the alignment of the nanocrystals.

Additional comparisons between medaka and zebrafish XRD data showed (see results per sample, shown as violin plots in Figure S3, Supporting Information) no significant difference in the average d‐spacing (approximately 6.88, *p* > 0.05). The values in the bones studied here fall within the range of nanocrystalline carbonated apatite reported for other bones (e.g., pike fish and mammalian bones: mouse and human).^[^ ^39^, ^40^, ^41^ ^]^

### 2.3 Elemental Distributions in Bones

**Figure 4:**
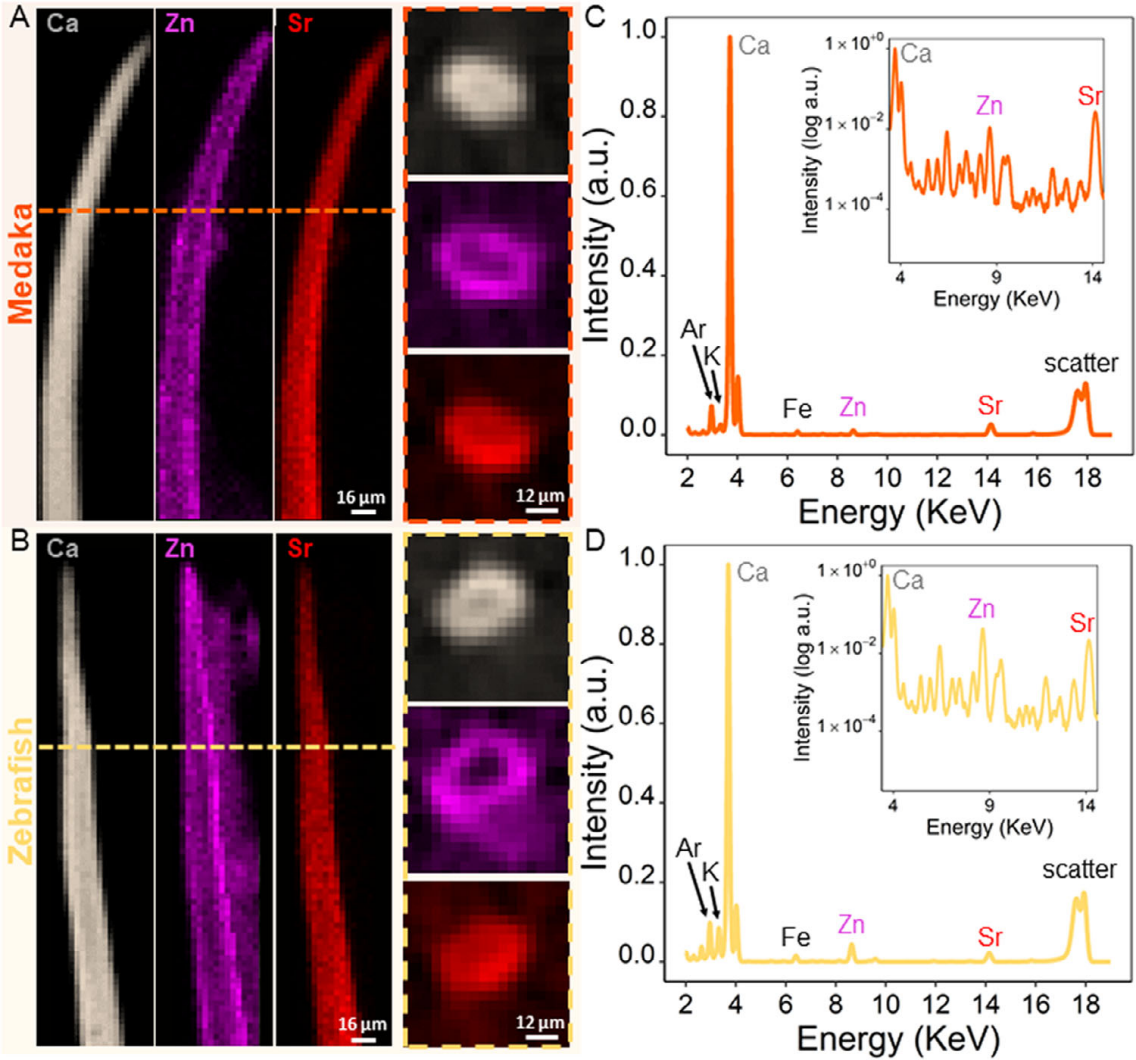
Examples of XRF 2D maps of Ca (gray), Zn (purple), and Sr (red) elements mapped along the length of spines of (A) medaka and (B) zebrafish bone with corresponding XRF tomography reconstructions. Medaka and zebrafish bone have similar elemental distributions along the spine. For both bone species, the Zn signal is higher at outer surface than in the bulk of the sample. Both Ca and Sr seem to be uniformly distributed. The XRF spectra of all measurement points in (C) medaka and (D) zebrafish bone show Ca, Sr and Zn peaks, with trace signals of other elements such as K (potassium) and Fe (iron) in the bone and Ar (argon) in the air.

The XRF spectra of all measurement points in **Figure** **4** show peaks corresponding mainly to Ca, Sr, and Zn in both medaka and zebrafish bones. There are also peaks corresponding to the elements Ar, K and Fe, although of low intensity (Figure 4C,D). These elemental compositions are very similar to those found in other vertebrate bones.^[^ ^34^, ^42^ ^]^ All 2D maps and reconstructed tomographic slices of Ca and Sr reveal uniform distributions, with Sr concentrations manifesting significantly lower counts than Ca. The 2D maps and corresponding XRF tomography reconstruction slices of Zn revealed higher concentrations on the outer sample surfaces than in the bone matrix.

**Figure 5:**
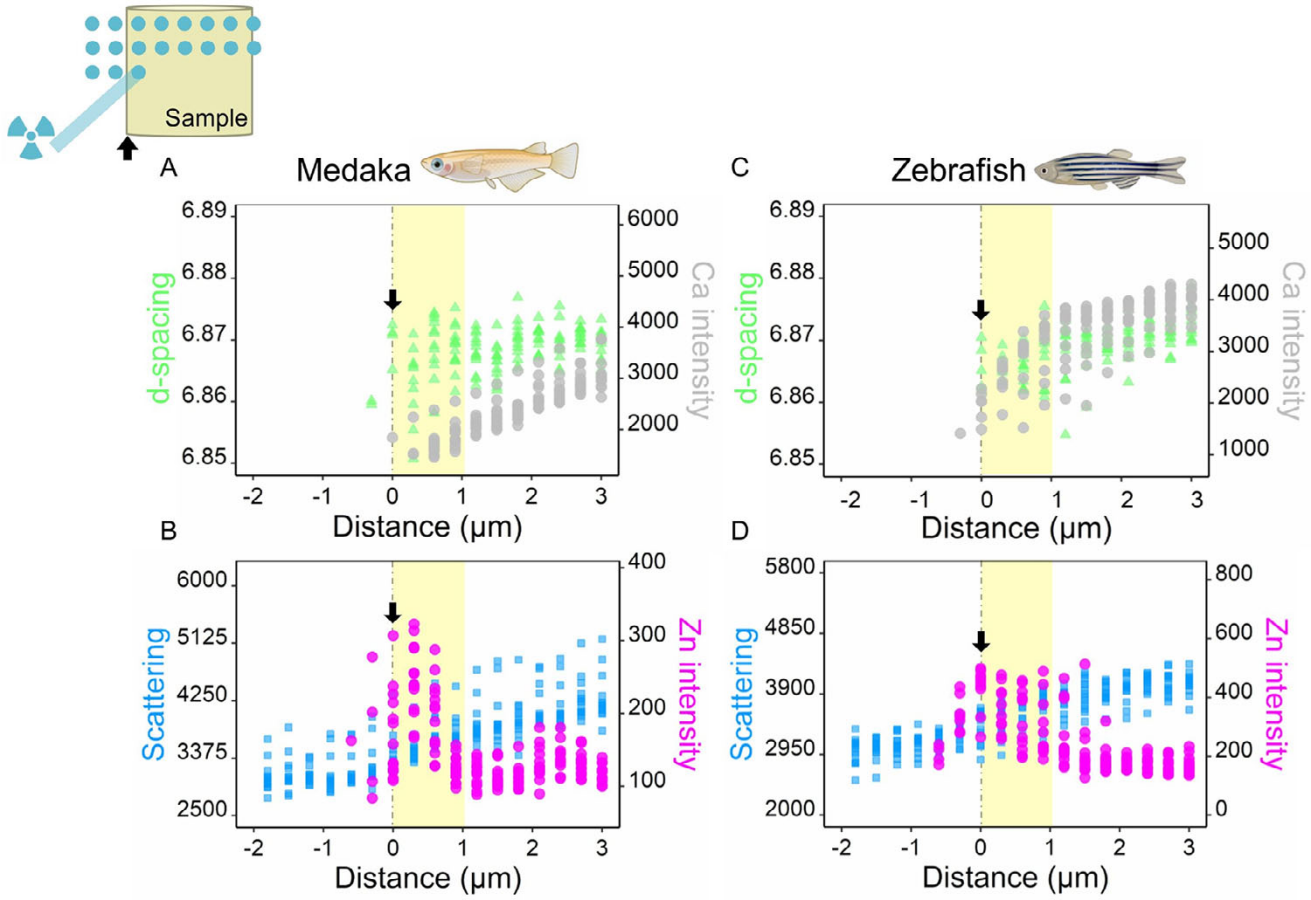
Distribution of Ca (gray), d‐spacing (green), Zn (purple), and elastic scattering (blue) at the edge of (A, B) medaka and (C, D) zebrafish bone. For both species, when scanning from outside the bone inward and approaching the bone edge (black arrow), the Zn signal appears before the Ca signal and d‐spacing value emerges from the XRD signal. With increasing diffuse scattering, the intensity of the Ca XRF signal and the value of d‐spacing increase from the bone edge inward. The rough edge regions (transition from soft tissue into bone material) of the bone are shaded in yellow.

High resolution scans with a beam and step size of 300 nm helped to better co‐localize the Zn and the bone nanocomposite at the edges of the samples. Figures S4 and S5 (Supporting Information) show results of 2D scans performed across several spines that reveal Ca, Zn, and d‐spacing. Due to a lower excitation energy used during these higher resolution scans (13 keV in the nanobeam setup), Sr was not detectable. However, the scans provide insight into the correlation between the nanocrystals and the chemical composition. For both bone types, Zn maps show a much higher concentration on the outer sample surfaces, clearly detected external to where Ca and apatite diffraction signals appear. Furthermore, the d‐spacing maps show lower c‐lattice values on the outer bone surfaces, as compared with nanocrystals at the center of the sample. The Ca distribution maps exhibit a gradient in intensity due to self‐absorption (Figure S6, Supporting Information). This is due to the fact that most of the emitted radiation (<4 keV) originating from Ca in the nanocrystals is absorbed in the bone such that regions that are nearer to the detector yield higher signals. Yet, all Ca maps show a concentration decrease at the sample edges until it vanishes, matching the crystal diffraction signature. Close‐up analysis of the measurements performed at the edges of the samples (**Figure** **5**, gray vertical line) reveals that there is a transition zone into bone. When scanning from outside inward, Zn was detected prior to the appearance of nanocrystal 002 Debye rings, with a relatively strong signal appearing only at the edges, corresponding to a transition zone. Zn is likely concentrated at the outermost surfaces of bone in the soft tissue lining the mineralized tissue, where it is predominantly present in protein‐bound or enzyme‐bound forms (e.g., in Alkaline phosphatases). It is likely to be incorporated into various matrix metalloproteinases, which are vital for preserving the extracellular matrix in soft tissues.^[^ ^43^, ^44^ ^]^ Figure 5 indicates that at the edges of bones there is a ≈1 µm thick transition zone from soft tissue into bone material (yellow shaded areas of Figure 5), where the first XRD signals are detected, scattering increases and the Zn peak diminishes. These results suggest that minimal, if any, Zn signal is located within the mineral nanocrystals. Ren et al. showed an increase in Zn content in synthetic apatites that corresponded to a decrease in the mineral c‐lattice parameter.^[^ ^45^ ^]^ That however is not the case for these bones, as we find no evidence that Zn partially replaced Ca in the apatite crystals in a way that could significantly affect the c‐lattice parameter. Similar observations were recently reported for bone in another fish species, pike (*Esox lucius*), where higher concentrations of Zn were found at the outer bone surfaces.^[^ ^41^ ^]^ Also in shark vertebrae, Zn is mainly concentrated on the outer surfaces.^[^ ^46^ ^]^ Such vertebrae are also composed of bioapatite, which is closely related to hydroxyapatite and organized similarly to bone. We conclude that bone surfaces feature Zn‐enriched rough edges forming a transition zone between the outer soft tissue and the bone material.

### 2.4 Are There Strain Gradients Across the Bones?

**Figure 6:**
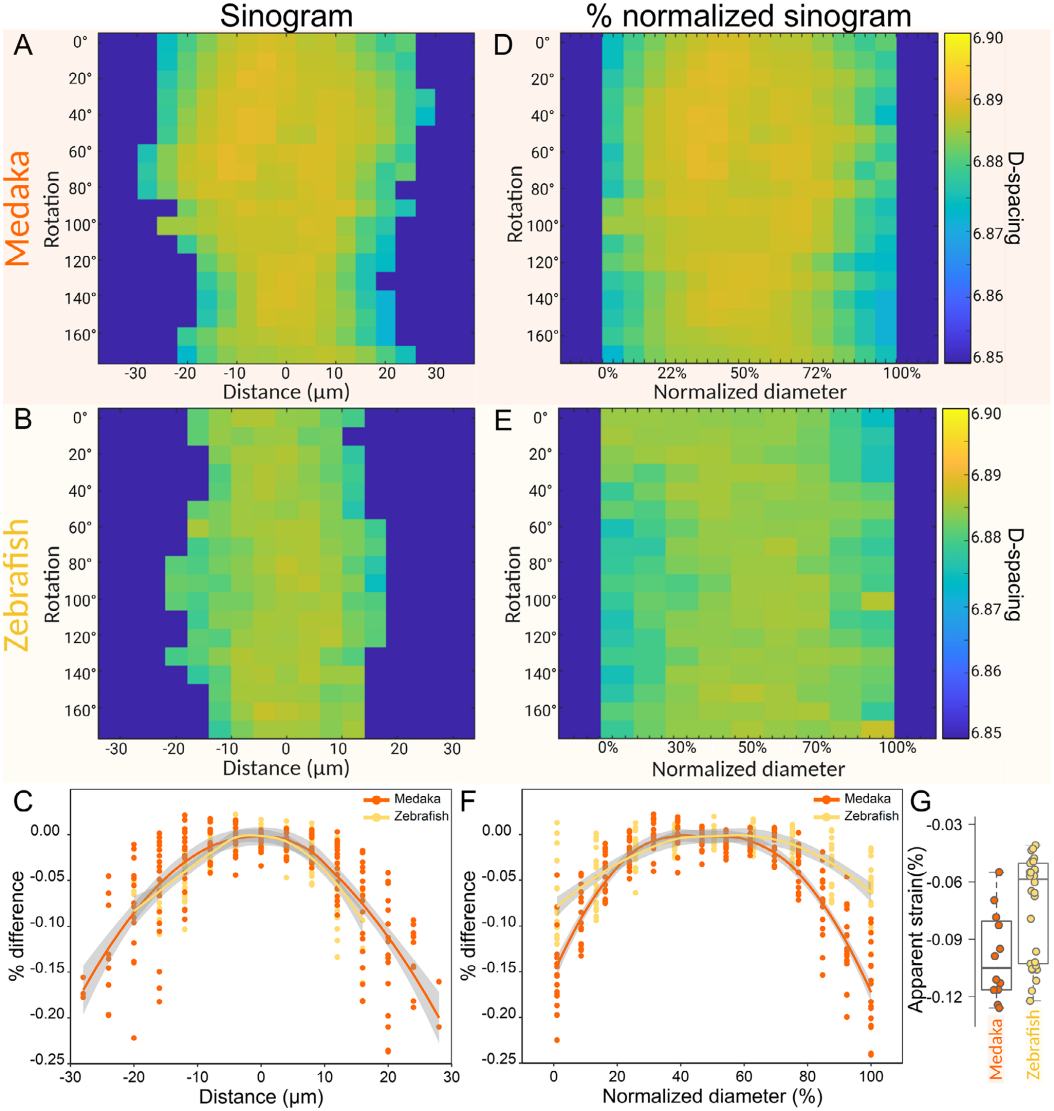
Examples of sinograms of d‐spacing distributions observed from different rotation angles for (A) medaka and (B) zebrafish bone. C) Scatter plots show the same data replotted as percent difference with respect to values from the sample center for both medaka and zebrafish. Trend lines across all angles of rotation indicate that the values at the center are higher than at the outer regions. A clearer picture emerges in the d‐spacing distribution of the normalized sinogram of (D) medaka and (E) zebrafish, where distance was normalized to the total sample width for better comparison between bone samples. F) Scatter plots of the percent differences at normalized distance shown similar trend with lower d‐spacing values at the outer regions compared to the bone center. The orange trend line of medaka has a greater decline compared to zebrafish trend line shown in yellow. G) Apparent strain distributions of medaka and zebrafish bones, are presented as boxplots on the right. Each strain value was calculated from a sinogram of different scans in several bones. Overall, medaka bone has an average apparent strain of −0.100 ± 0.02 and zebrafish has an average strain of −0.071 ± 0.03. The boxplots show that medaka bone has higher apparent residual strain than zebrafish bone which is statistically significantly different between the two fish species (*p* < 0.05).

Tomographic XRD data revealed that the c‐lattice parameter of the apatite nanocrystals consistently differs between the central regions and the outer edges of all spine bones. **Figure** **6**A,B plots examples of d‐spacing results from line scans of two samples measured along different rotation angles, plotted as sinograms (line position versus angle). For quantitative comparison, the d‐spacing sinograms are additionally replotted, normalized as % of the bone width. Common to all angles shown in Figure 6D,E is that there is always higher d‐spacing (yellow color) at the center regions and a lower d‐spacing (blue color) near the outer bone margins. This becomes strikingly apparent in the data plotted as a percentage difference with respect to values at the sample center (trend lines of Figure 6C,F). We observe a clear difference in the c‐lattice parameter, as quantified by fits to the data. This indicates compression in the nanocrystals of the bone material near the outer surfaces suggesting that those crystals are deformed to experience “apparent strain”. Detailed trend lines of the sinogram data are provided in Figure S6 (Supporting Information), and all show apparent compressive strain across all bone samples. Box plots (Figure 6G) suggest that in both medaka and zebrafish spines, there is higher compressive residual strain in the outer bone flanks. These plots of percentage differences (between the center and flanks) reveal that medaka bone exhibits an average apparent strain of −0.10%, while zebrafish bone exhibits an average apparent strain of −0.07%. Thus, the distribution of the apparent strains revealed a statistically significant difference (p < 0.05) between the two bone species, such that the apparent residual strain in medaka is about 30% higher than that in zebrafish bone. These findings raise the question of whether the compressive residual strains observed in these bones are an intentional design strategy to enhance mechanical performance or merely a product of the mineralization process. We hypothesize that the osteocytes within the central region of zebrafish bone contribute to strain relaxation in zebrafish since bone porosity is less able to retain stress in the surrounding extracellular matrix.

### 2.5 Residual Strain as a Nano‐Strengthening Mechanism of Bones

**Figure 7:**
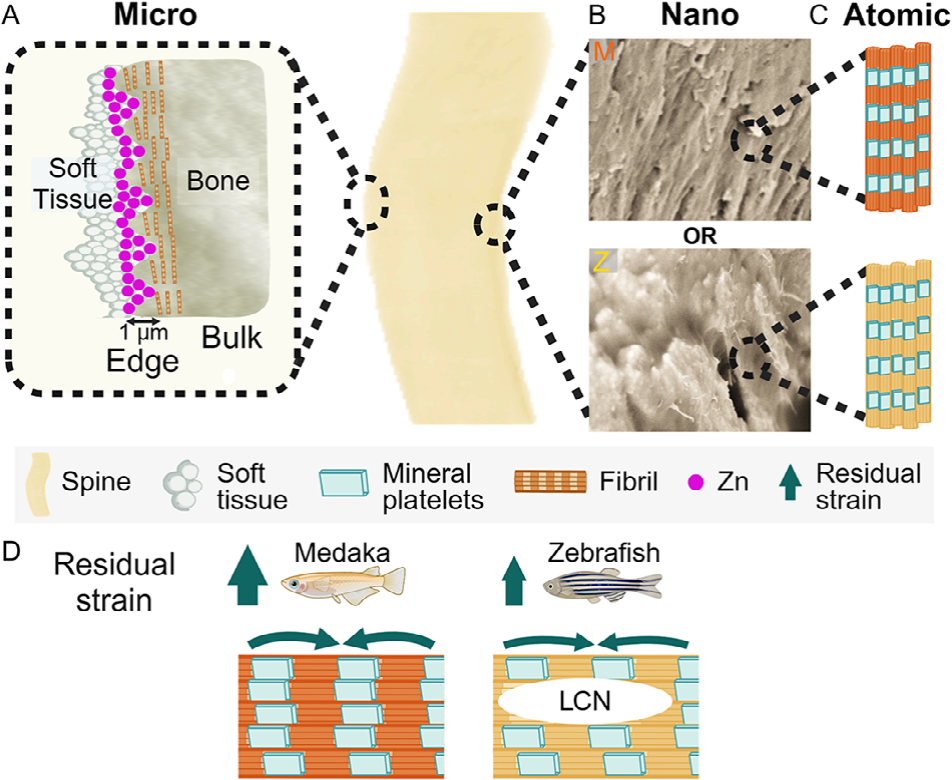
Schematic representation of our proposed bone model in the fish spines: A) At the microscopic scale, bone surfaces exhibit a transition zone between the outer soft tissue and the bone material. Compression of mineralized collagen fibrils at these surfaces generates residual strains. B) At the nanoscale, anosteocytic medaka bone is distinguished from osteocytic zebrafish bone by the absence of LCN voids. In osteocytic bone, voids may limit the amount of compression that the nanocrystals can stand C) At the atomic scale, both bone types appear similar in chemical composition, with nanocrystals in mineralized collagen fibrils predominantly aligned along the principal bone axis. D) Compressive residual strain within the bone tissue enhances toughness, with anosteocytic bone exhibiting higher residual strain compared to osteocytic bone.

We propose that in both fish species, the bone texture leads to material strengthening through compressive residual strain on the outer bone flanks, which makes use of the tight attachment between hydroxyapatite nanocrystals and collagen nanofibers.^[^ ^27^, ^47^, ^48^ ^]^ The proposed model is depicted schematically in **Figure** **7** and operates at the nanometer composite level. This model acts against crack initiation on the outer surfaces of the spines, likely providing a safety margin against catastrophic failure due to repeated load cycles (e.g., during swimming).^[^ ^49^ ^]^

In both species, nanocrystals are aligned with the collagen fibrils along the principal axis of the bone. Compressive residual strains on the outer bone edges also act along this principal axis, so that when muscles pull between the spines, the bone material bends but experiences reduced stress levels in tension. This structural design can potentially create a fracture resistant material that helps prevent failure when external loads are applied. A study on bovine bone demonstrated that residual strains are present in the longitudinal direction, aligning with the mineralized collagen fibrils.^[^ ^26^ ^]^ More recently, Ping et al. showed that intrafibrillar mineralization generated collagen fibril contraction.^[^ ^47^ ^]^. They suggested that the collagen fibers that are tightly connected to the hydroxyapatite nanocrystals lead to mineral compression. Various bones exhibit natural strain gradients from the center to the outer surfaces. A recent study of pike fish revealed that in wet bones, nanocrystals are pre‐loaded near the bone outer surfaces by ≈0.15% in compression.^[^ ^41^ ^]^ After dehydration, residual strains remain entrapped in the bone. Our own work shows exemplary XRD line scans of medaka and zebrafish bones, measured under wet conditions (presented in Figure S7, Supporting Information). Medaka bone exhibits residual strains of −0.12 %, whereas the zebrafish bone shows almost no apparent residual strains. These findings only hint at a difference in residual strain between the two species when measured wet. However, it is important to note that the experimental setup for 2D line scans under wet conditions differed from the tomographic scans performed under dehydrated conditions, for technical reasons. To avoid damage to the bones we included significant amounts of soft tissue in these samples which may have mechanically loaded the bones to somewhat modify the degree of crystal deformation. XRD tomography of such hydrated bones will require complex in situ experimental setups, to be pursued in future studies.

In the results shown here, we found no indications that the chemical composition of bones has any substantial impact on deforming the apatite nanocrystals (that might be interpreted as residual strain), because the distribution of elements is more or less uniform throughout the bone tissue. At the bone edges, however, there is a higher concentration of Zn, which appears to be connected to the soft tissue surrounding the bone, as illustrated schematically in Figure 7A. As a result, Zn cannot extensively replace Ca in the apatite structure, and thus it likely does not alter the c‐lattice parameter of the mineral nanocrystals. An important observation from our data is that the residual strain is almost 30% higher in anosteocytic bones. These bones lack osteocytes and are therefore free from LCN porosity. This suggests that bone microarchitecture can influence residual stress distributions within bone. Medaka bones may have fewer structural defects (lack of LCN porosity, as shown in Figure 2), enabling them to better distribute and accommodate residual stresses across the mineralized collagen fibrils. As a result, these bones exhibit a higher strain gradient between the center and outer surfaces compared to zebrafish bones. Lattice strains are mainly caused by collagen fibrils compression. Residual strains in the nanocrystals on the outer flanks of the fish spines are the result of the longitudinal arrangement of collagen fibers, which exert pulling forces on the bone nanostructure. Though the presence or absence of LCN porosity does not seem to affect the alignment of the nanocrystals in bone tissue, it may have an impact on the manner in which residual strain operates. We hypothesize that any residual deformation of crystals in the bone matrix surrounding lacunae voids may be partially released. Intriguingly, the majority of the lacunae voids are situated in the central regions of zebrafish spines (see lacunae distribution evaluation in Figure S8, Supporting Information). This suggests that many nanocrystals present in the center region may be unable to resist deformation compared to those in the bone surface regions. On the other hand, the LCN voids also act as stress raisers, areas within the bone tissue where stress concentrates under external loading. It is therefore possible that a lower level of residual stress may be sufficient for protecting the integrity of zebrafish bone, due to its ability to repair itself. Interestingly, at the macroscopic scale, it has been shown that anosteocytic bones fail at higher strains than mammalian bones do. Atkins et al. found that anosteocytic bones in billfish exhibit a yield strain comparable to that of mammals, ranging from 0.7% to 0.8%, with failure occurring at strains greater than 3–4%.^[^ ^9^ ^]^ Thus, compressive residual strains integrated into the bone nanocomposite might be an evolutionary mechanism to enhance bone strength and delay failure, potentially favoring the emergence of anosteocytic bone in advanced teleost fish.

Residual stress significantly affects the mechanical properties of materials, influencing and often enhancing their strength.^[^ ^31^, ^50^ ^]^ In engineering materials, these stresses can arise from nonuniform deformations during manufacturing due to external influences such as mechanical, thermal, or phase changes.^[^ ^51^, ^52^, ^53^ ^]^ Depending on their nature, residual stresses can be either beneficial or harmful.^[^ ^51^ ^]^ For instance, compressive residual stresses generally improve fatigue resistance by delaying crack initiation and growth, while tensile residual stresses are undesirable and impact both material performance and lifespan.^[^ ^51^ ^]^ Residual strains are also present in various biological systems, such as mollusk shells, brittle star skeletons, and tooth dentin.^[^ ^31^, ^54^, ^55^, ^56^, ^57^ ^]^ Such strains may arise from intracrystalline organic and inorganic inclusions or from interactions between organic and inorganic components near intercrystalline interfaces.^[^ ^31^ ^]^ In many materials, such a mechanism increases fracture toughness and therefore can protect against damage.^[^ ^29^, ^30^ ^]^ We posit that compression of the mineral nanocrystals by the strong collagen fibrils in the fish spines creates residual strains that increase fracture toughness and protect the bone against damage.^[^ ^47^ ^]^ Compressive residual strain may be particularly important for anosteocytic bone that lacks the osteocyte‐mediated remodeling process.^[^ ^5^, ^6^ ^]^ However, further fracture‐toughness analysis is needed to better understand the resistance to crack‐growth and toughness of anosteocytic and osteocytic bone material. Furthermore, elucidating how stresses become embedded in the mineral phase of biological tissues can help the development of innovative engineering designs that incorporate toughening strategies.

## 3 Experimental Section

### 3.1 Materials

Spinal columns from nine adult medaka and eleven adult zebrafish were used. The fish were discarded from a swim training research project, comparing differences in bone growth, described elsewhere.^[^ ^7^ ^]^ Fish experiments were approved by the ethics committee of the Hebrew University of Jerusalem (permit MD‐16‐14844‐3). In accordance with guidelines, healthy fish were euthanized using an overdose of tricaine methane‐sulfonate. The vertebral column of each fish, which was cryopreserved at −80 °C, was extracted using a stereo‐dissection microscope, during which most of the soft tissue was carefully removed before isolating the 5^th^ vertebra, avoiding breakage of the spines. Samples were dehydrated in ascending graded series of ethanol solutions (50%, 75%, and 100%), and the hemal spine of each vertebra was mounted upright on a pin holder and fixed using a light‐curing transparent resin.

### 3.2 PCE µCT

**Figure 8:**
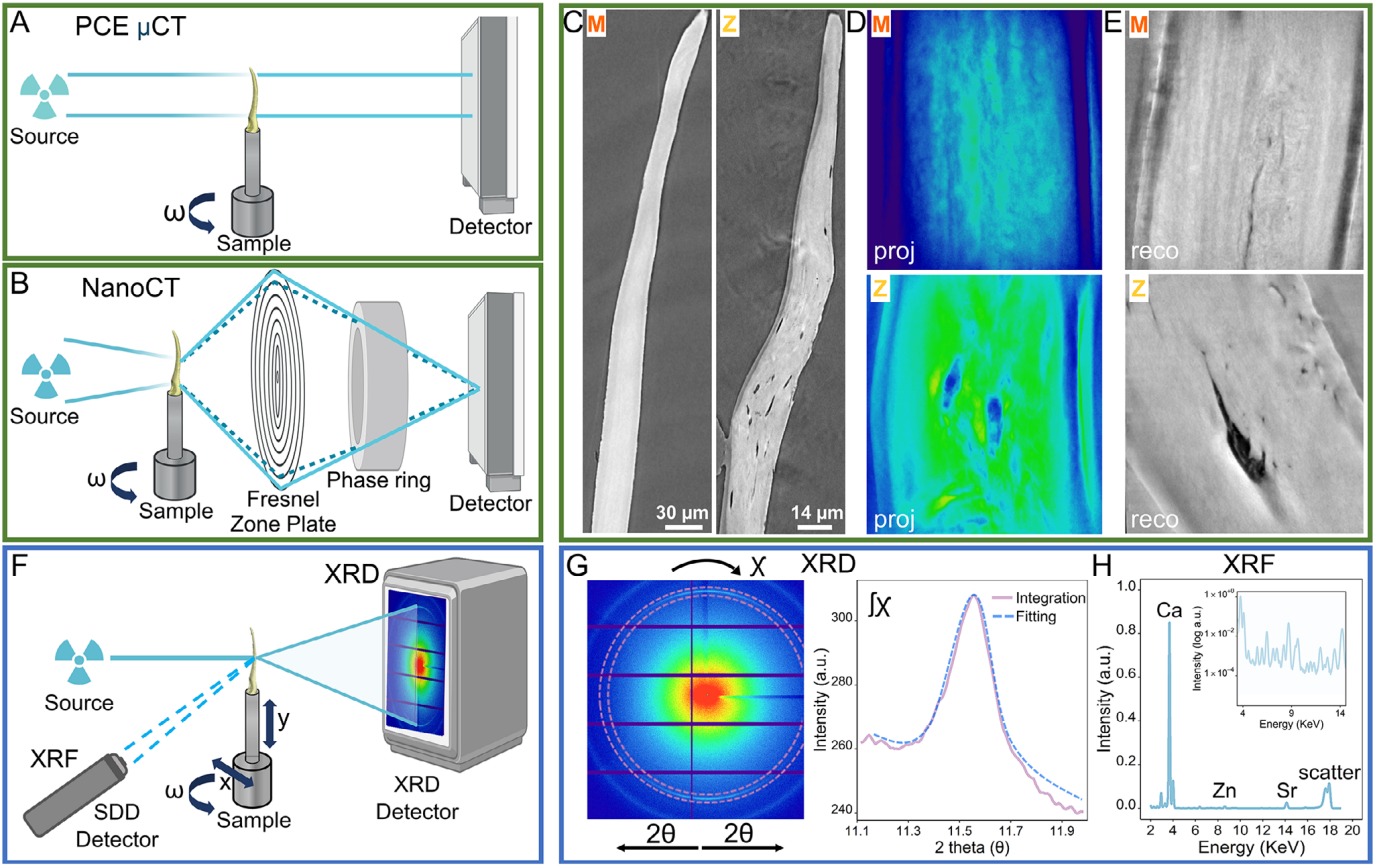
Overview of all the imaging techniques used to analyze medaka and zebrafish bones: A) Illustration of the PCE µCT setup at the ANATOMIX beamline, SOLEIL, France, including the X‐ray source, detector, and sample. The spine of each fish was mounted and placed on the rotation stage. A parallel monochromatic X‐ray beam is transmitted through the sample and is recorded by the detector as a 2D projection image. Projection images are collected as the sample is rotated at increments through ω over a range of 180°. B) Schematic representation of the experimental setup of the Zernike nanoCT at the transmission X‐ray microscope (TXM) of ANATOMIX. In this setup the X‐ray beam is targeted onto the sample by a condenser lens, and then is split into two components. One component (undiffracted wave, dotted brown line) passes through a Fresnel zone plate whereas the other (diffracted wave, dark blue line) magnifies the sample image. The undiffracted wave is phase‐shifted by the phase ring on the path to the camera system. The detector images the overlap resulting in interference patterns arising from these two waves. For tomographic imaging, a series of radiographs is collected similar to the PCE µCT setup. C) Examples of PCE µCT reconstruction slices of medaka and zebrafish bones versus D) Zernike nanoCT radiographs and E) respective typical reconstruction slices. Note the increased contrast and resolution when using Zernike nanoCT as compared to PCE µCT. NanoCT reconstruction slices are coupled with the appearance of halo and shade‐off artifacts that are clearly identifiable at the edges of the sample. F) Schematic of the XRD and XRF experimental setup conducted at the P03 beamline, DESY, Germany. Spine samples were probed by a focused X‐ray beam while the sample stage moved in x and y directions and rotated ω in increments, spanning 180°. The diffraction patterns were collected on a direct‐illumination hybrid pixel detector whereas fluorescent X‐rays emitted by the sample were collected by an XRF SSD detector. G) Example of a typical diffraction pattern. Azimuthal (χ) integration of the 002 Debye ring (dotted purple region) as a function of scattering angle (2θ) creates 1D diffraction intensity profiles (purple line) that can be fitted by a Voigt function (dotted blue line). H) Example XRF spectra of the same measurement points (inset shows magnified log scale plot). The peaks corresponding to the elements Ca, Zn, and Sr are identified in the spectra plot.

Three medaka and four zebrafish spines were scanned at the ANATOMIX beamline at Synchrotron SOLEIL, Saint‐Aubin, France. Spines were imaged using a µCT setup with inline phase contrast enhancement employing a 325 nm pixel size.^[^ ^58^ ^]^ **Figure** **8**A exemplifies the PCE µCT experiment performed at 17 keV and 200 ms exposures. A detector with a Hamamatsu Orca Flash 4 V2 2048×2048 CMOS‐based camera and lutetium aluminum garnet single‐crystal scintillator, coupled to each other via commercial microscope optics (Mitutoyo 20x, numerical aperture 0.4), were used. A total of 2000 projections were collected for each sample, imaged by rotation over 180°.

### 3.3 NanoCT

The same spines scanned using the PCE µCT setup were also imaged with the Zernike‐nanoCT setup at the TXM of the ANATOMIX beamline, SOLEIL.^[^ ^59^ ^]^ The experimental setup of Zernike‐nanoCT imaging at the TXM branch, illustrated in Figure 8B, comprises a beam‐shaping condenser lens in front of the sample, a Fresnel zone plate behind the sample, a set of concentric Zernike phase rings to induce phase contrast, and a beam stop before the condenser to block the direct X‐ray beam. The same detector as for the PCE µCT scans was used. The TXM setup is described in more detail by Scheel et al.^[^ ^59^ ^]^ A region 150 µm below the tip of each spine was selected and imaged at a pixel size of 47 nm. Scans were conducted at 10 keV using 1s exposures and a total of 500 projections were collected for each sample.

### 3.4 XRF and XRD Data Collection

The spines of three medaka and three zebrafish were scanned using the MINAX nanofocus endstation on beamline P03 of PETRA III (DESY, Hamburg). A schematic illustration of the experimental setup is shown in Figure 8F. Each sample was mounted on a translation and rotation stage for translation in the x, y in plane, to measure lateral points on the 1475 × 1679 pixel PILATUS 2M (Dectris) detector, as well as rotated 180° around the sample axis. The experiment was performed using a 4 × 4 µm^2^ beam at an X‐ray energy of 18 keV, collecting XRD and XRF spectra (Figure 8F) on lines scanning multiple slices along each sample with exposure times of 0.5 s. After each line‐scan, the sample was rotated with incremental angles of ≈10° to obtain 2D sinograms of x versus ω. Additional 2D maps of the full spines at the 0° position were also collected with an exposure time of 0.8 s. In total, 3500 – 4700 points were collected for each tomography scan. Each scattering pattern corresponds to X‐ray diffraction along the beam path through the sample, as shown in Figure 8G. The fluorescence spectra were recorded by an XRF detector (Amptek X123) placed at 45° to the incident beam, as illustrated in Figure 8H. In a follow‐up experiment, additional line scans across the spines were collected with a beam with a diameter of 300 nm. One medaka and two zebrafish were scanned using an X‐ray energy of 13 keV and exposure time of 0.5 s to create high resolution 2D maps of the spines and in particular the outer bone regions. In addition, two medaka and two zebrafish were scanned under wet conditions using an X‐ray energy of 19.8 keV.

### 3.5 Data Analysis

#### 3.5.1 CT Data Reconstruction

PCE µCT datasets were reconstructed using the standard data processing pipeline available at the beamline, based on a user frontend and pre‐processing code developed at SOLEIL and, as the reconstruction backend, the software PyHST2.^[^ ^60^ ^]^ Projection radiographs were normalized by flat and dark correction using reference images acquired at the beginning and end of each scan. For the nanoCT scans, a median filter with a radius of two pixels was applied to normalized projections to reduce noise. Tomographic reconstructions for those scans were performed using the back‐projection method implemented in NRecon (NRecon 1.7.1.0, Bruker micro‐CT, Kontich, Belgium). The reconstructed nanoCT volumes were cropped and binned using Fiji,^[^ ^61^ ^]^ resulting in an effective pixel size of 94 nm (binned) and true resolution of 130 – 150 nm. Each nanoCT dataset comprised approximately 420 cross‐section slices (each 512 × 512 pixels) that make up the 3D volume. Examples of nanoCT radiographs and micro‐ and nanoCT reconstructed slices of medaka and zebrafish spines revealing bone, pores, and edge‐enhanced halo and shade‐off artifacts are shown in Figure 8E as well as in Videos S3 and S4 (Supporting Information).

#### 3.5.2 CT Data Segmentation and Registration

PCE µCT and nanoCT volumes were segmented and co‐aligned by 3D registration using Dragonfly (Dragonfly 2021.3; Object Research Systems, Montreal, Quebec, Canada).^[^ ^62^ ^]^ Semantic segmentation was performed using the deep learning (DL) libraries following the protocol proposed by Silveira et al.^[^ ^20^ ^]^ The DL Sensor3D model was trained to identify small and large internal pores as well as the outer geometry of the bone. Volume quantification of the bone and the LCN pores in the zebrafish spines was achieved using the Dragonfly “multi‐ROI analysis” module. For each zebrafish sample, the volume of the spine region scanned with nanoCT and the volume of the corresponding full‐length spine scanned at µm‐resolution were 3D registered. The nanoCT volume was approximately placed within the µCT volume at the height where the high resolution scan was performed, and 3D registration using the mutual information method was performed to superimpose both volumes. Volume registration was performed to know precisely identifying which section of the spine was scanned at higher resolution.

#### 3.5.3 XRF Processing and Line‐Scan Reconstruction

XRF spectra collected with the µm and nm‐sized beams were analyzed using in‐house scripts in Fiji and MATLAB (V.2022) with tomographic reconstructions performed with NRecon. From all XRF spectra, the Ca, Sr, and Zn elements peaks were processed to create 2D maps for each element by custom MATLAB scripts. A sinogram was created for each tomographic line‐scan and element. Due to mechanical instabilities of the rotation stage, the lateral relation between the sample and the rotation center was not constant for some of the angles within entire 180° angular scans. Therefore, making use of the known dilute homogeneous distribution and low self‐absorption of Sr, the sinogram lines were co‐aligned around the center. This approach was used to determine and correct axis positions to overcome the lateral motor misalignments. For every angle, a cross correlation and digital shift were applied in MATLAB to create a well‐defined rotation center required for reconstruction. Once the rotation center was corrected for Sr, the same shifts were applied to the Ca and Zn sinograms. All sinograms were thereafter normalized by the background noise level, converted into projections, and reconstructed using the back‐projection method in NRecon.

#### 3.5.4 XRD Processing

XRD data were processed to provide information about the apatite nanocrystal c‐lattice parameter (d‐spacing) and orientation within the bone matrix. Calibration was performed using lanthanum hexaboride powder (LaB_6_) to refine the sample‐to‐detector distance, beam center, rotation, and camera tilt angles.

##### d‐Spacing Analysis

To extract the atomic planes of the c‐lattice parameter of the nanocrystals of the bony carbonated apatite, the XRDUA software package was used.^[^ ^63^ ^]^ For each scanned line, all diffraction patterns were azimuthally integrated to create 1D diffraction intensity profiles as a function of the scattering angle 2θ. Background scattering was corrected, and the intensity profiles were fitted using a Voigt function with custom MATLAB code based on the Faddeeva package. The c‐lattice parameter of bone apatite was calculated from the known relations between d‐spacing and the Bragg condition:

(1) $$d=\frac{n\times \lambda }{2\times sin(\theta )}$$

where λ is the X‐ray wavelength, n is an integer corresponding to the order of diffraction and θ is the angle between the incident X‐ray and crystal planes.^[^ ^64^ ^]^ The XRD data collected along lines with different rotation angles around the spines defined slices, and the c‐lattice parameters were used to create 2D sinograms corresponding to the different perspectives and positions in each slice. Non linear motor shifts (as described in the XRF processing Section 3.5.3) were corrected based on the Sr sinograms.

##### Apparent Strain Analysis

The bony nanocrystals c‐lattice is oriented primarily along the collagen axis in the mineralized collagen fibrils of the bone nanocomposite. Previous work had shown that collagen was well able to compress tightly coupled nanocrystals of apatite.^[^ ^41^ ^]^ To explore this effect in the fish spines, the XRD d‐spacing sinograms of the 002 peak were used for the strain analysis, with apparent lattice strains in the apatite crystals calculated as:

(2) $$\varepsilon =\frac{d-d_{0}}{d_{0}}$$

where d denotes the d‐spacing at each point, and d_0_ refers to a reference point in the sample center. Apparent strains across each slice in each sample were determined by comparing the d‐spacing at the center and the outer edges of the bone, plotted in the sinograms for each rotation angle. To allow comparisons between samples with differing diameters (sample diameters were in the range of 35 to 55 µm), the sample thickness was normalized to 100%. A regression model was then fitted to each rotation angle in the normalized sinogram. These fits were then used to precisely determine the d‐spacing at the center and outer edges of each angle and each sample, and the c‐lattice parameter served to calculate apparent strain.

##### Orientation Analysis

For each XRD pattern collected at 18 keV, the 002 apatite peak is situated between 11.4° and 11.7° on the 2θ‐scale. All diffraction patterns were radially integrated over 36 regions of the Debye rings, in 10° steps, using the Pyfai package of Python.^[^ ^65^ ^]^ This led to 1D data exhibiting two peaks separated by 180°. The resulting intensity profiles were fitted using double‐peak Gaussian function. The peak positions of the complete 002 rings, corresponding to the preferred orientation of the mineral crystals, were determined for each angle and measurement point. Based on the intensity differences between the peak areas with a preferred orientation it was possible to calculate the degree of preferred orientation within each bone. Sinograms based on orientation and degree of preferred orientation (DoD) values of each line scan were created taking into consideration shifts due to non linear motor movements, corrected based on the Sr sinograms of each slice (see Section 3.5.3).

### 3.6 Statistical Analysis

Statistical analyses were performed using RStudio (V1.3, RStudio, PBC, Boston, MA, USA). The Wilcoxon signed‐rank test was used to compare the d‐spacing and apparent strain values between medaka and zebrafish bones. P‐values <0.05 were considered statistically significant.

## 4 Conclusion

Our work shows the similarities and material differences between anosteocytic and osteocytic bones using multiple high‐resolution materials characterization techniques. Beyond the visible difference in the presence (or lack) of osteocytes, the results revealed that medaka and zebrafish bones have similar elemental compositions and spatial distributions of hydroxyapatite nanocrystals within the bone matrix. NanoCT coupled with Zernike phase contrast revealed details regarding bone texture, giving hints about the collagen fibril orientation with respect to bone geometry. XRD showed that in both medaka and zebrafish, the mineralized collagen fibrils in the bones generate favorable residual strains on the outer surfaces of the spines. In addition, medaka bone has a significantly higher residual strain as compared with zebrafish bone. These findings revealed that medaka bone, devoid of LCN porosity, provides a greater residual strain gradient between the center and outer surfaces of the bone compared to zebrafish. Compression residual stress on the outer flanks of the bones may increase bone resistance to damage (e.g., preventing cracking while repeatedly loaded during swimming) and may thus help prevent failure. Here, we highlighted the similarities and differences between anosteocytic and osteocytic bone nanocomposite materials that may help to understand the adaptation of fishbones to different environmental conditions.

## Conflict of Interest

The authors declare no conflict of interest.

## Supporting information

Supporting Information

Supplemental Video 1

Supplemental Video 2

Supplemental Video 3

Supplemental Video 4

## Data Availability

The data that support the findings of this study are available from the corresponding author upon reasonable request.
