# Supplementary material for: Nanocrystal Compressive Residual Stresses: A Strategy to Strengthen the Bony Spines of Osteocytic and Anosteocytic Fish
Source: Adv Sci (Weinh). 2025 Apr 11;12(20):2410617. doi: 10.1002/advs.202410617 (PMC12120745; doi:10.1002/advs.202410617)
Supplement: Supplementary file 1 — Supporting Information [file ADVS-12-2410617-s003.pdf]

ADVANCED SCIENCE
Open Access

Supporting Information

for *Adv. Sci.*, DOI 10.1002/advs.202410617

Nanocrystal Compressive Residual Stresses: A Strategy to Strengthen the Bony Spines of Osteocytic and Anosteocytic Fish

*Andreia Silveira, Anton Davydok, Christina Krywka, Mario Scheel, Timm Weitkamp, Claudia Fleck, Ron Shahar and Paul Zaslansky**

1 | SUPPLEMENTARY INFORMATION

TABLE S1. Morphometric parameters of the lacunae and canaliculi of each zebrafish bone sample scanned with Zernike-nanoCT.

Sample	BV (μm^3)	Lc.N	Lc.TV (μm^3)	Lc.V (μm^3)	Lc.TV/ BV (%)	Ca.N	Ca.TV (μm^3)	Ca.V (μm^3)	Ca.TV/ BV (%)	LCN.TV (μm^3)	LCN.TV/ BV (%)
1	429×10^2	7	581	83	0.22	61	93	13.30	1.36	674	1.57
2	652×10^2	12	1063	89	1.63	76	405	20.26	0.62	1470	2.26
3	363×10^2	4	216	54	0.60	39	85	6.56	0.23	301	0.83
4	382×10^2	4	221	55	0.58	38	147	9.80	0.39	368	0.96
Average	457×10^2	6.8	398	70	0.76	54	305	12.48	0.65	703	1.40

BV=bone volume; Lc.N=lacunae number; Lc.TV=lacunar total volume; Lc.V=lacunar average volume; Lc.TV/BV=lacunar porosity; Ca.N=canaliculi number; Ca.TV=canalicular total volume; Ca.V=canalicular average volume; Ca.TV/BV=canalicular porosity; LCN.TV=LCN total volume; LCN.TV/BV=LCN porosity.

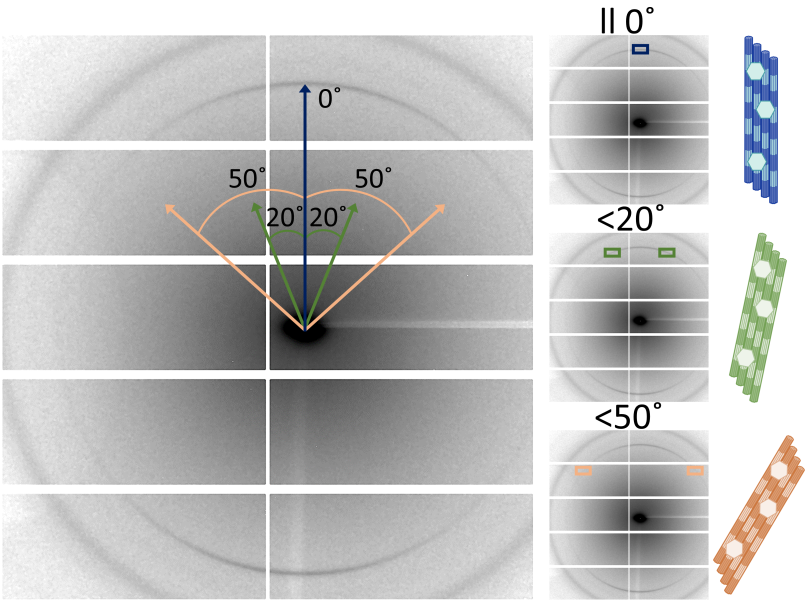

FIGURE S1. Example of a diffraction pattern used to calculate the orientation of hydroxyapatite crystals with respect to the vertical axis (on-principal-axis) of the pattern center. Rectangle regions around the upper 002 arc were selected at specific angles: 0 ° on-principal-axis (blue rectangle), 20 ° off to the right and left (green rectangles) and 50 ° off to the right and left (orange rectangles). From all the diffraction patterns, the average scattering intensity within each rectangle was calculated, and the corresponding sinogram was added to reconstruct the average scattering intensity at 0 °, 20 °, and 50 °. Ratios between 0 °/20 ° and 0 °/50 ° were measured to provide hints about the mineral distribution within zebrafish and medaka bones.

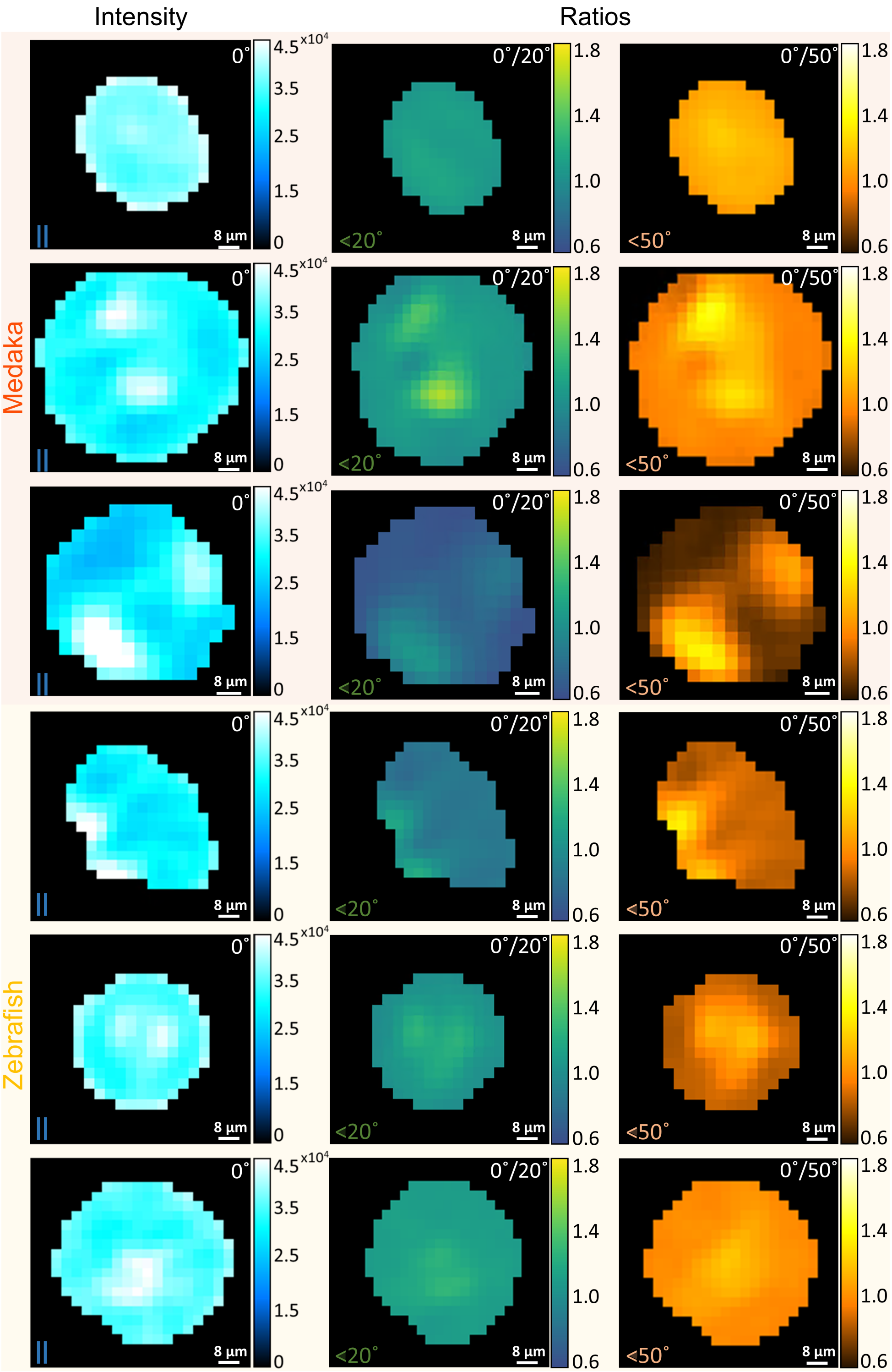

FIGURE S2. Reconstructed tomographic slices of each medaka and zebrafish bone sample showing the mineral intensity at on-principal-axis (0 °) region and the ratios of on-principal-axis versus 20 ° (0 °/20 °) and 50 ° (0 °/50 °) off-principal-axis The average intensity is ~ 1.5 – 1.8 times stronger along the principal axis than at 20 ° or 50 ° off the principal axis for both medaka and zebrafish bones.

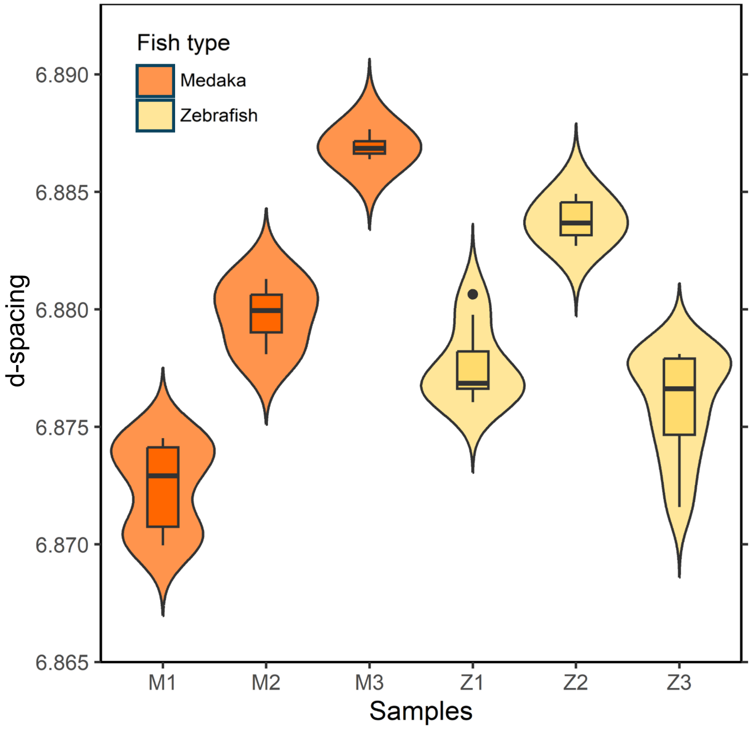

FIGURE S3. D-spacing values per sinogram are presented as violin plots for all medaka and zebrafish bone samples. The wider sections of the violin plots indicate a higher density of data points, while the narrower sections indicate lower densities. The central box plots within the violin plots displayed the median d-spacing per sample. Medaka bone has an average d-spacing of 6.88 ± 0.007. Similarly, zebrafish bone has an average d-spacing of 6.88 ± 0.004. There is no significant difference between bone species ($p > 0.05$).

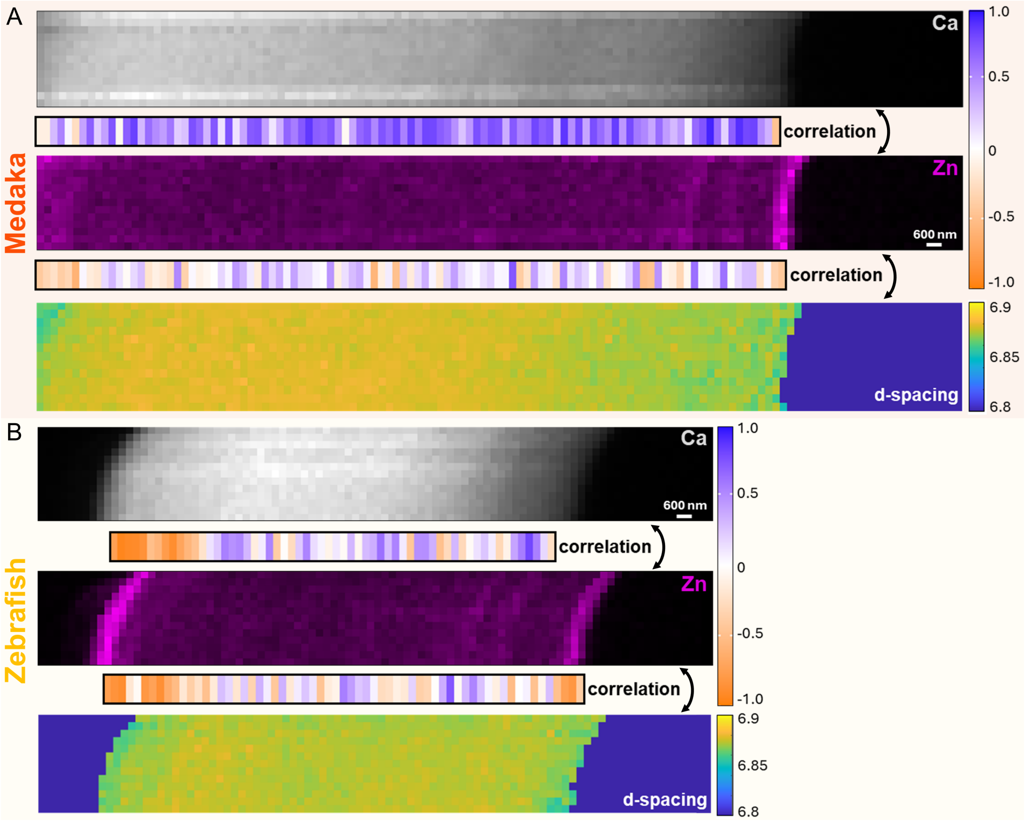

FIGURE S4. Comparisons using correlation bars between XRF 2D maps of Ca (gray) and Zn (purple), and XRD d-spacing map at 300 nm resolution scanned from spine regions of (A) medaka and (B) zebrafish bone. Ca distribution maps exhibit a uniform distribution with a stronger signal on one side due to higher proximity to the detector (self-absorption by the sample in regions further away from the detector). The Zn signal is notably much higher on the outer surfaces of both bones. The d-spacing maps show higher values at the center than on the outer bone edges. The Zn and Ca signals appear to be positively correlated for most of the inner part of bones but negatively (inversely) correlated near the outer bone edges. Zn and d-spacing are mostly negatively correlated on the flanks of bones.

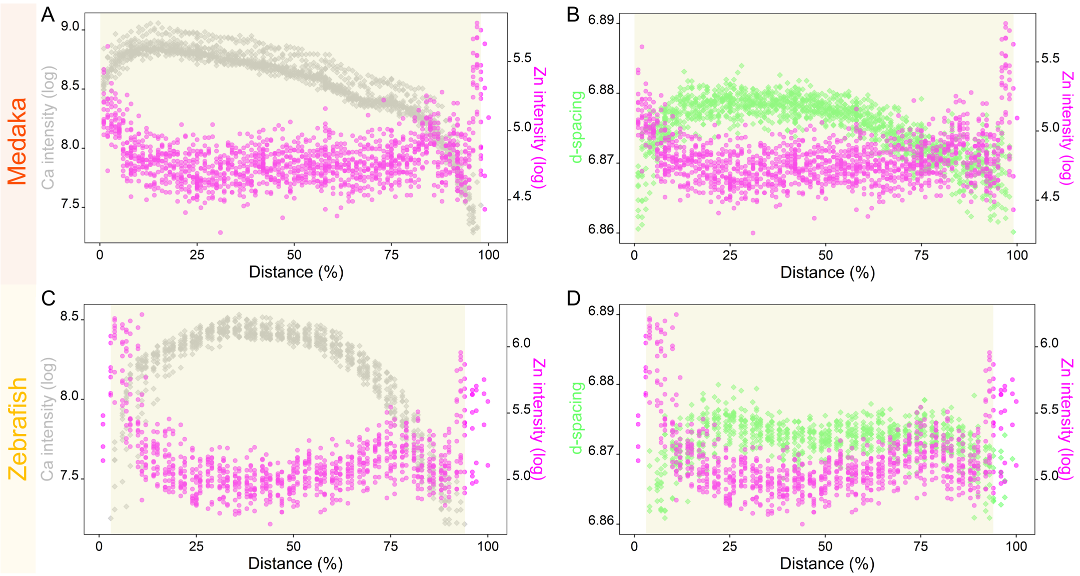

FIGURE S5. (A) Ca vs Zn and (B) d-spacing vs Zn values from XRF and XRD data distributed along medaka spine width, that was normalized to the total sample thickness. The distribution of Zn has a strong peak at the margins of medaka bone. While Ca values as well as the d-spacing values diminished at the boundaries of the samples. Similarly, (C) Ca vs Zn and (D) d-spacing vs Zn plots of zebrafish bone show a strong Zn peak and a decrease in Ca and d-spacing signal at the bone edges. Both medaka and zebrafish bones are marked with a beige rectangle.

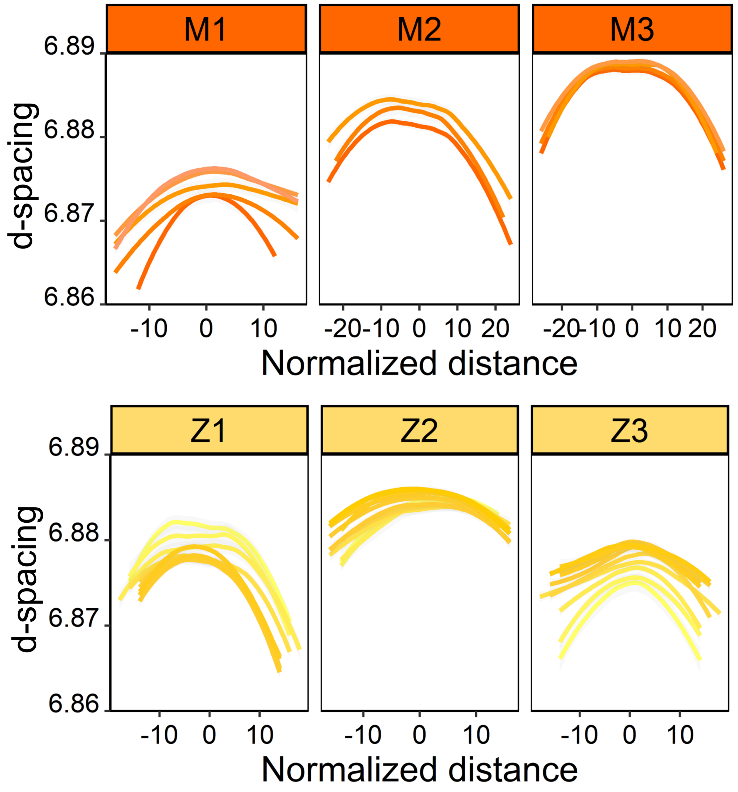

FIGURE S6. Trend lines of the scatter plots of d-spacing values at a normalized distance for each sinogram of each medaka and zebrafish sample. Both medaka and zebrafish have similar line trend, d-spacing values are lower at the outer regions compared to the bone center. The maximum d-spacing at the center region varies more within medaka than in zebrafish samples.

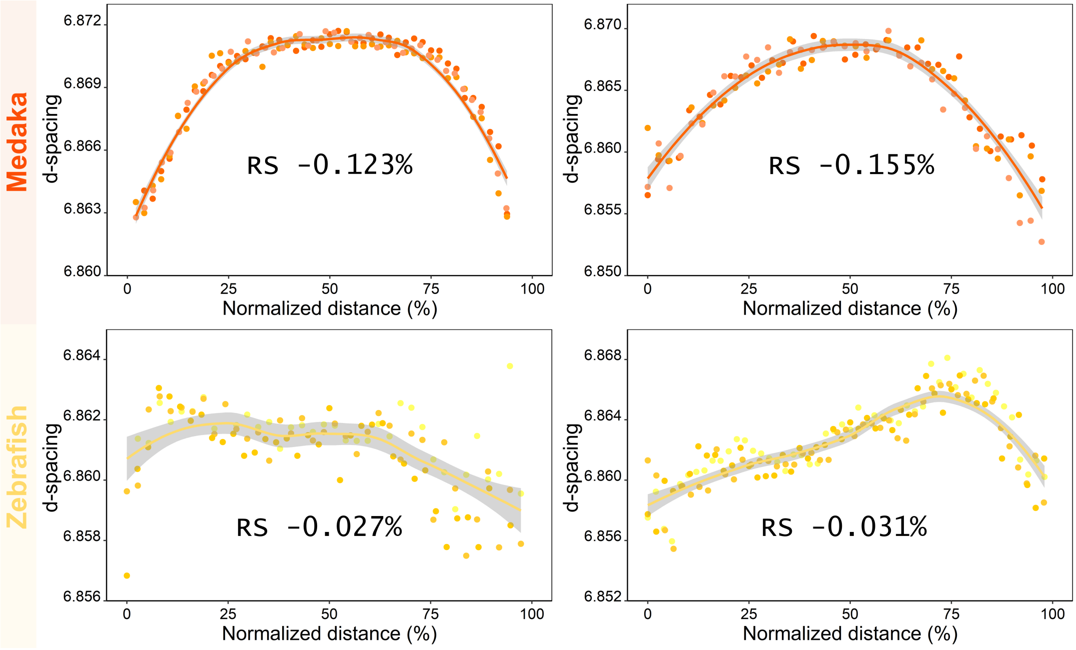

FIGURE S7. Scatter plots display the d-spacing distribution along the spines of (A, B) medaka and (C, D) zebrafish samples under wet conditions. Line scans were performed at 19.8 keV. In medaka bone, trend lines reveal higher d-spacing values at the center compared to the outer regions, with apparent residual strains (RS) of - 0.123% and - 0.155%. In contrast, zebrafish trend lines show a much smaller decline, corresponding to lower residual strains of - 0.027% and - 0.031%. These preliminary results suggest that medaka bone exhibits higher residual strain values than zebrafish bone under wet conditions.

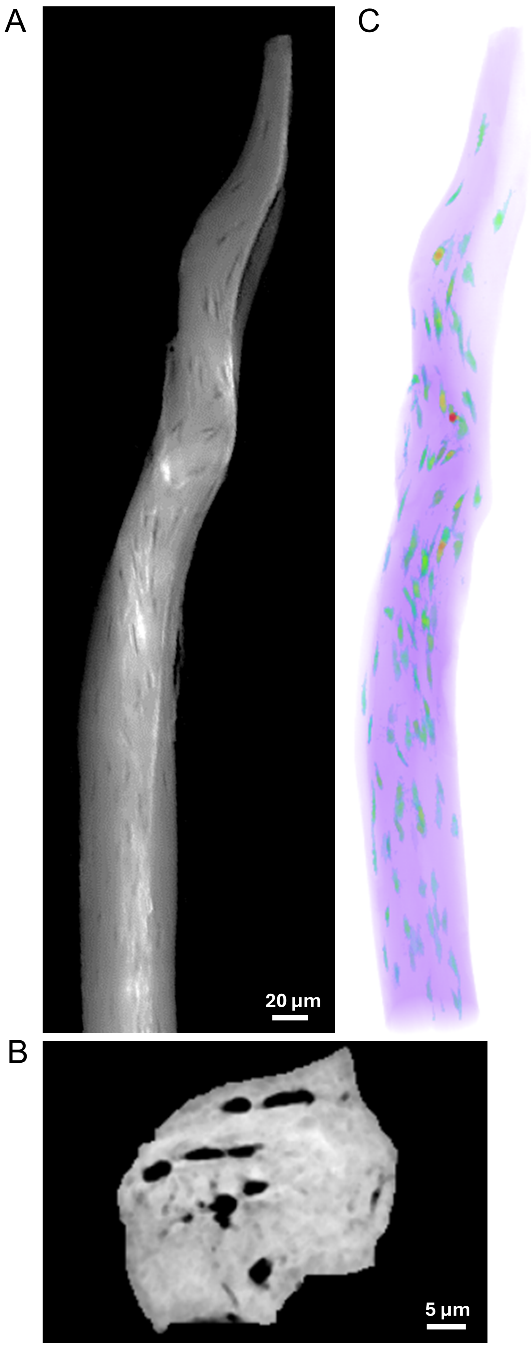

FIGURE S8. (A) Longitudinal and (B) vertical sections of a z-stack projection of zebrafish bone, showing the lacunae voids distribution along the spine. (C) 3D render of the lacunae voids within zebrafish spine. Most of the lacunae seem to be concentrated in the central region of the spine.
